# Stimulation of Macrophages with the β-Glucan Produced by *Aureobasidium pullulans* Promotes the Secretion of Tumor Necrosis Factor-Related Apoptosis Inducing Ligand (TRAIL)

**DOI:** 10.1371/journal.pone.0124809

**Published:** 2015-04-13

**Authors:** Koji Kawata, Atsushi Iwai, Daisuke Muramatsu, Shiho Aoki, Hirofumi Uchiyama, Mitsuyasu Okabe, Sumio Hayakawa, Akinori Takaoka, Tadaaki Miyazaki

**Affiliations:** 1 Aureo Science Co., Ltd., Sapporo, Hokkaido, Japan; 2 Aureo Co., Ltd., Kimitsu, Chiba, Japan; 3 Division of Signaling in Cancer and Immunology, Institute for Genetic Medicine, Hokkaido University, Sapporo, Japan; 4 Department of Probiotics Immunology, Institute for Genetic Medicine, Hokkaido University, Sapporo, Japan; University of Pittsburgh School of Medicine, UNITED STATES

## Abstract

A β-glucan produced by *Aureobasidium pullulans* (AP-PG) is consisting of a β-(1,3)-linked main chain with β-(1,6)-linked glucose side residues. Various β-glucans consisting of β-(1,3)-linked main chain including AP-PG are believed to exhibit anti-tumor activities, and actually, anti-tumor activities of AP-PG in mice have been demonstrated. In this study, we demonstrate that stimulation with AP-PG induces TRAIL expression in mouse and human macrophage-like cell lines. TRAIL is known to be a cytokine which specifically induces apoptosis in transformed cells, but not in untransformed cells. The expression of TRAIL mRNA after stimulation with AP-PG was increased in RAW264.7 cells, Mono Mac 6 cells, and macrophage-differentiated THP-1 cells. The mRNA expression of TNF-α and FasL is only weakly increased after stimulation with AP-PG. The induction activity of TRAIL by curdlan, a bacterial β-glucan, was very similar to that by AP-PG in RAW264.7 cells, but weaker in macrophage-differentiated THP-1 cells. Activation of caspases was found in HeLa cells after treatment with the supernatant of cultured medium from AP-PG-stimulated Mono Mac 6 cells, and was inhibited by the anti-TRAIL neutralizing antibody. These findings suggest that the stimulation with AP-PG effectively induces TRAIL in macrophages, and that it may be related to apoptosis induction of tumor cells.

## Introduction

A polysaccharide, β-(1,3)-D-glucans (β-glucans) consisting of a β-(1,3)-linked main chain are known to be an immune stimulator [1–3]. Administration of β-glucans produced by a variety of organisms, such as mushrooms, yeasts, and fungi, are believed to exhibit a variety of beneficial effects. Due to this, these β-glucans are commercially available and consumed as food supplements. Among the various proposed efficacies, the most prominent effect of β-glucans would be its anti-tumor activity. Actually, several anti-tumor drugs containing β-glucan as the main compound, such as Krestin [4], Picibanil [5], Lentinan [6], and Sizofiran [7], have been developed and are used in the treatment of cancers. Although, in principle, these β-glucan-based drugs are used in combination with other chemical anti-tumor drugs, it has been demonstrated that β-glucans enhance the anti-tumor activity of chemical anti-tumor drugs through the activation of immunity to eliminate tumor cells in clinical trials.

Black yeast, *Aureobasidium Pullulans* produces a water soluble β-glucan (AP-PG) in growth medium under certain conditions [8, 9]. AP-PG is characterized by being more highly branched with β-(1,6)-D-glycosidic linked glucose residues than β-glucans derived from other organisms [9, 10]. Overall, the effects of AP-PG in mammals are assumed to be the same as those of β-glucans derived from other organisms, and actually, anti-tumor, anti-allergy, and anti-infectious disease effects of AP-PG have been identified under experimental conditions [11–15].

It is speculated that the induction of apoptosis of tumor cells plays an important role in the anti-tumor activity of β-glucan. Apoptosis is classified as type I programmed cell death, and is characterized by morphological changes such as cell shrinkage, chromatin condensation, and DNA fragmentation [16]. Apoptosis is an important physiological mechanism to remove injurious cells from the body. Therefore, strategies for induction of apoptosis specific to tumor cells are thought to be important in the development of anti-cancer drugs. Several extracellular cytokines are related to the transmission of cell signals for induction of apoptosis, and especially, death ligands belonging to the tumor necrosis factor (TNF) superfamily are assumed to be central to the signal transduction for apoptosis induction. Here, TNF-α, FasL (Fas ligand), and TRAIL (TNF-related apoptosis inducing ligand) are well known death ligands, and TRAIL, the most recently identified molecule among these ligands, is thought to be crucial for the tumor killing. As TRAIL has been identified as an apoptosis inducing ligand which specifically induces apoptosis to transformed cells but not to normal cells [17], there have been attempts to develop novel drugs utilizing the characteristics of TRAIL.

The aim of this study is to propose a possible mechanism on the anti-tumor activity of AP-PG. This report shows that stimulation with AP-PG has the potential to induce TRAIL-dependent apoptosis through an activation of the TRAIL expression in macrophages. Stimulation with AP-PG induces TRAIL in mouse and human macrophage-like cell lines, RAW264.7 cells [18], MONO MAC 6 cells [19], and macrophage-differentiated THP-1 cells [20, 21]. The induction activity of TRAIL by AP-PG was compared with curdlan, a bacterial β-glucan. The results show that both curdlan and AP-PG similarly induce TRAIL expression in RAW264.7, whereas TRAIL induction activity with curdlan in macrophage-differentiated THP-1 cells is apparently weaker than that with AP-PG. In addition, the supernatant of cultured medium from Mono Mac 6 cells stimulated with AP-PG activates caspases-8 and -9 in HeLa cells, and these activations of the caspases are inhibited by a neutralizing antibody against TRAIL. These findings suggest the possibility that the stimulation with β-glucan is involved in the induction of apoptosis to tumor cells through the expression of TRAIL in macrophages.

## Results

### Expression of TRAIL is increased after stimulation with the β-glucan produced by *A*. *pullulans* (AP-PG) in RAW264.7 cells

It has been reported that β-(1,3)-D-glucans branched with the β-(1,6)-linkage including AP-PG take part in immune stimulation activity and also exhibit anti-tumor activity [11, 12]. The death ligands belonging to the TNF superfamily, TNF-α, FasL, and TRAIL, are known to be important in the killing of tumors through the induction of apoptosis [22, 23]. To assess the immune stimulation activity of AP-PG on the expression of these death ligands, we performed real-time RT-PCR analysis using a mouse macrophage-like cell line, RAW264.7 cells, and found that TRAIL mRNA was significantly increased after the stimulation with AP-PG.

**Fig 1:**
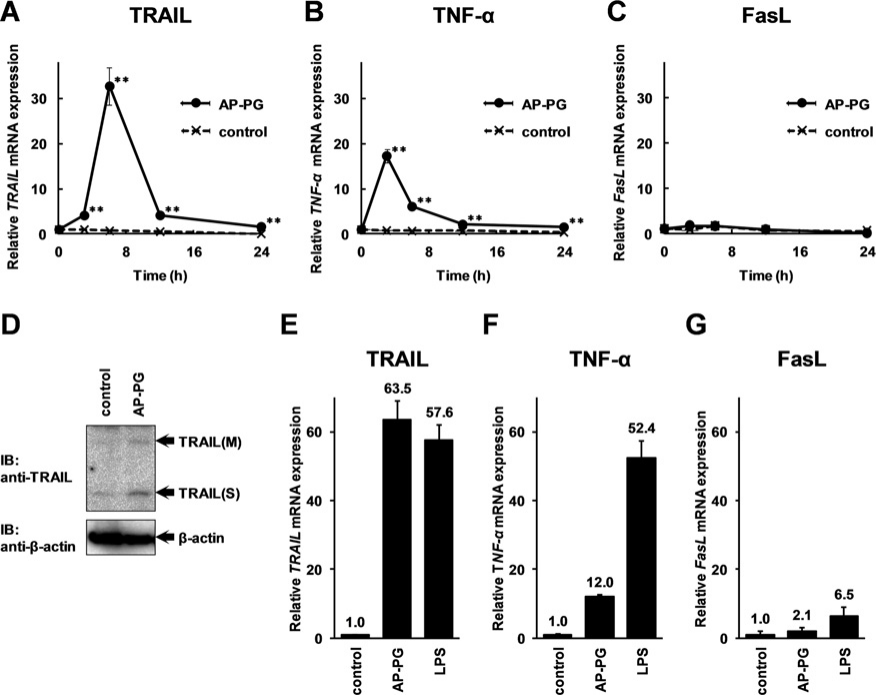
TRAIL mRNA expression is increased after the stimulation with AP-PG in a mouse macrophage-like cell line, RAW264.7 cells. (A-C) 6 × 10^5^ of RAW264.7 cells cultured in a 35 mm culture dish RAW264.7 cells were stimulated with 100 μg/ml of AP-PG. After 0, 3, 6, 12, and 24 hours, the cells were harvested, and the total RNAs isolated from the cells were subjected to real-time RT-PCR analysis using a specific primer sets for TRAIL (A), TNF-α (B) and FasL (C) genes. Data represent relative expression values compared with the mRNA expression at 0 hour after normalizing with the GAPDH mRNA expression. Error bars indicate standard deviations calculated by three independent experiments. Double asterisk (**) indicate p<0.01. (D) RAW264.7 cells were stimulated with 100 μg/ml of AP-PG for 24 hours, and then the whole cell extracts from the cells were subjected to Western blotting analysis using a specific antibody against TRAIL. The anti-β-actin antibody was used for the loading control. (E-G) RAW264.7 cells were stimulated with AP-PG or lipopolysaccharide at a concentration of 100 μg/ml. After 6 hours, the total RNA isolated from the cells was subjected to real-time RT-PCR analysis using specific primer sets for TRAIL (E), TNF-α (F), or FasL (G). The data represent relative expression amounts compared with that of the control cells after normalizing with the GAPDH mRNA expression. The values in the graphs indicate the means, and error bars indicate standard deviations calculated from three independent experiments.

The results of time course analysis comparing with the expression of other death ligands indicated that the increment of TRAIL mRNA expression after stimulation with AP-PG was greater than that of TNF-α and FasL mRNAs (Fig 1C–1E). The results show that expression of TRAIL mRNA is significantly increased from 3 hours and peaked at 6 hours in RAW264.7 cells after stimulation with AP-PG. In addition, the results of Western blotting analysis using a specific antibody against TRAIL indicated that both soluble and membrane bound forms of TRAIL protein were induced in RAW264.7 cells after the stimulation with AP-PG (Fig 1D). Similarly, the expression of TRAIL mRNA was increased after the stimulation with lipopolysaccharide (LPS), a well known immune stimulator (Fig 1E). These results compare favorably with a previous report which demonstrated the involvement of LPS in TRAIL induction in macrophages [24]. However, TNF-α mRNA was also more strongly increased after the stimulation with LPS than with AP-PG (Fig 1F). The results indicate differences in selectivity on the induction of death ligands by the stimulation between AP-PG and LPS. In addition, FasL mRNA indicated higher levels of expression after the stimulation with LPS than after stimulation with AP-PG (Fig 1G). These results demonstrate that the stimulation of RAW 264.7 cells with AP-PG effectively induces TRAIL.

### Stimulation with AP-PG also induces TRAIL in human macrophage-like cell lines

**Fig 2:**
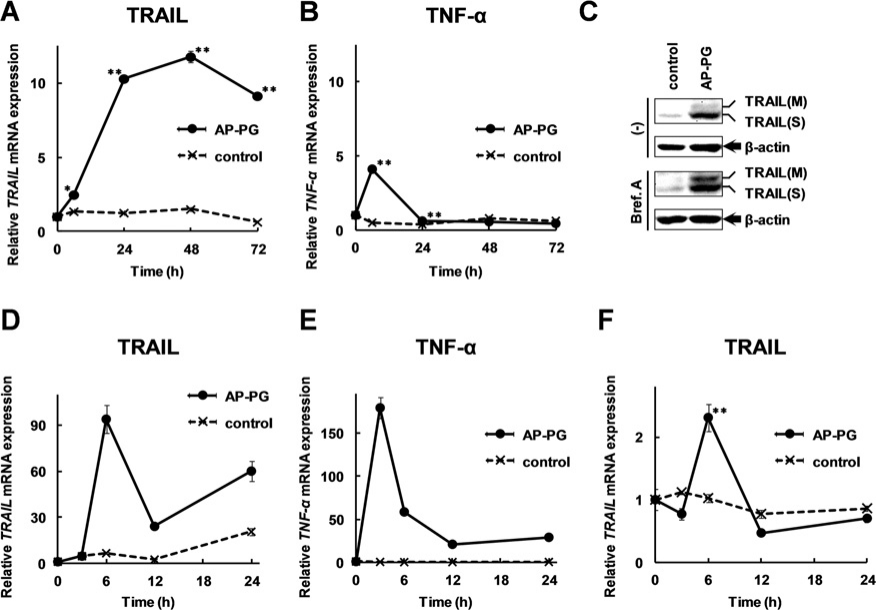
Stimulation with AP-PG effectively induces TRAIL mRNA in a human macrophage-like cell line, Mono Mac 6 cells. (A, B) 6 × 10^5^ of Mono Mac 6 cells cultured in a 35 mm culture dish were stimulated with AP-PG at a concentration of 100 μg/ml. After 0, 6, 24, 48, and 72 hours, the cells were harvested, and the total RNAs isolated from the cells were subjected to real-time RT-PCR using a specific primer sets for TRAIL (A) and TNF-α (B) genes. (C) Mono Mac 6 cells were stimulated with 100 μg/ml of AP-PG for 21 hours, and then the cells were treated with 10 μg/ml of Brefeldin A (Bref. A). After an additional 3 hour incubation period, the whole cell extracts prepared from the cells were subjected to Western blotting analysis using a specific antibody for the TRAIL protein. An anti-β-actin antibody was used for loading the controls. (D, E) THP-1 cells (6×10^5^ cells in a 35 mm culture dish) differentiated into macrophages using PMA were simulated with 100 μg/ml of AP-PG for 0, 3, 6, 12, and 24 hours, and then the total RNAs isolated from the cells were subjected to real-time RT-PCR analysis using specific primer sets for TRAIL (D) or TNF-α (E) genes. (F) 1 × 10^5^ of normal human monocytes were differentiated into macrophages, and then stimulated with 100 μg/ml of AP-PG. After 0, 3, 6, 12, and 24 hours, the cells were harvested, and the total RNAs isolated from the cells were subjected to real-time RT-PCR analysis using a specific primer sets for TRAIL mRNA. Data represent relative expression amounts compared with that of control cells after normalizing with the GAPDH mRNA expression. The values in the graphs indicate the means, and error bars indicate standard deviations calculated from three independent experiments.

We confirmed that the TRAIL induction also took place in human macrophage-like cell lines. Initially, using Mono Mac 6 cells which are known to exhibit mature macrophage phenotypes [19], the TRAIL expression after the stimulation with AP-PG was investigated. As shown in Fig 2A, AP-PG stimulation also significantly induced TRAIL mRNA in Mono Mac 6 cells, although the response to the stimulation with AP-PG was slower than that of RAW264.7 cells. The TRAIL mRNA expression is significantly increased after 6 hours and peaked at 48 hours after the stimulation with AP-PG. Similar to the results with RAW264.7 cells, expression of TNF-α mRNA was weaker than that of TRAIL mRNA (Fig 2B), and FasL mRNA was not detected in Mono Mac 6 cells in our experimental conditions (data not shown). Western blotting analysis using a specific antibody against TRAIL demonstrated that the stimulation with AP-PG effectively induces a soluble form of TRAIL in Mono Mac 6 cells (Fig 2C, upper panels). Further, to investigate whether TRAIL protein is secreted from the cells after stimulation with AP-PG, we used Brefeldin A, an inhibitor for protein transport from the endoplasmic reticulum to the golgi apparatus. The Mono Mac 6 cells were stimulated with AP-PG for 21 hours, and then the cells were treated with Brefeldin A for 3 hours. The results show that the membrane bound form, TRAIL (M) induced after stimulation with AP-PG was accumulated in the cells after the treatment with Brefeldin A (Fig 2C, compare the first and third panels). These results indicate that the TRAIL protein induced after stimulation with AP-PG would be secreted as soluble form, TRAIL (S) from the cells.

Next, we investigated whether the TRAIL induction is also found in a human monocyte derived cell line, THP-1 cells differentiated into macrophages. In macrophage-differentiated THP-1 cells, the time-course of TRAIL mRNA induction after stimulation with AP-PG, its expression level was peaked at 6 hours, and almost same pattern was observed in that of RAW264.7 cells. As shown in Fig 2D, the results show that the TRAIL mRNA expression was significantly increased after stimulation with AP-PG. On the other hand, TNF-α mRNA strongly increased in macrophage-differentiated THP-1 cells 3 hours after treatment with AP-PG (Fig 2E) when compared with the increase occurring in RAW264.7 cells (Fig 1).

Finally, TRAIL mRNA expression in normal human macrophages after stimulation with AP-PG was investigated. As shown in Fig 2F, TRAIL mRNA expression in the normal human macrophages differentiated from peripheral monocytes was significantly increased after stimulation with AP-PG. These results indicate that stimulation with AP-PG can induce TRAIL mRNA in macrophages.

### Induction of TRAIL in Mono Mac 6 cells stimulated with AP-PG is involved in the induction of apoptosis of tumor cells through the activation of caspases

Activation of the caspase cascade is crucial for the initiation of apoptosis [25, 26]. The death receptor-mediated signaling pathway is responsible for activating the caspase-8 in response to stimulation with death ligands, and also involved in the caspase-9 activation through the activation of JNK (c-Jun N-terminal kinases) followed by an increment in mitochondrial permeability [27–29]. In addition, activation of caspase-8 is also involved in the caspase-9 activation through the proteolytic activation of Bid (BH3 interacting-domain death agonist) which antagonizes the anti-apoptotic function of Bcl-2 and Bcl-xL.

**Fig 3:**
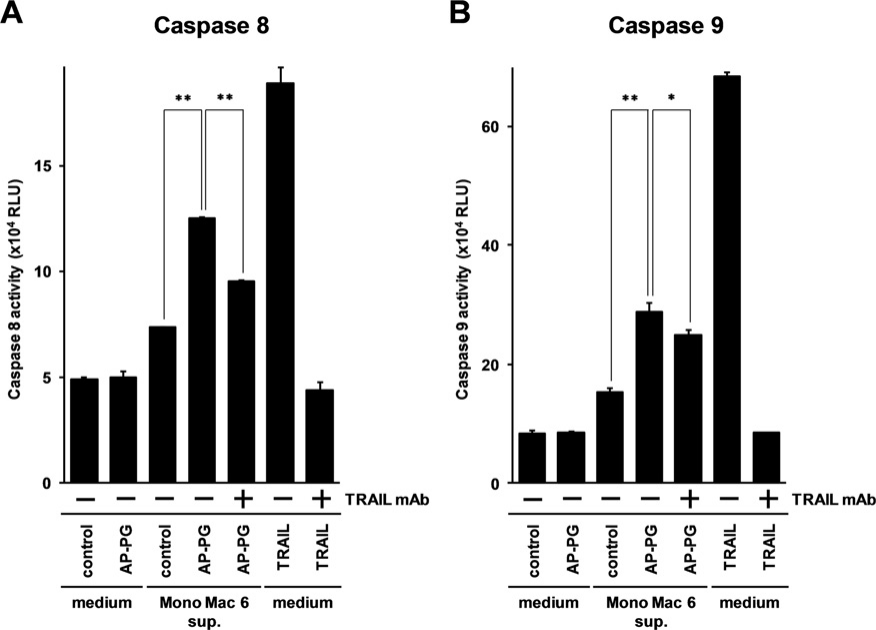
TRAIL secreted by the Mono Mac 6 cells after the stimulation with AP-PG is able to induce apoptosis to HeLa cells. (A) Mono Mac 6 cells (3 × 10^5^ cells/ml) were stimulated with AP-PG at a concentration of 100 μg/ml. After 48 hrs, the supernatant of the cultured medium was collected, and HeLa cells (2.5 × 10^4^ cells in 24 well plate) were cultured in the supernatant with 100 ng/ml cycloheximide for 3 hrs. After the incubation period, the caspase-8 activity was measured using Caspase Glo Assay kit (Promega). 1 μg/ml neutralizing antibody for TRAIL was added to the supernatant for monitoring the dependence of TRAIL on the caspase-8 activation. HeLa cells incubated in the growth medium with 100 μg/ml AP-PG were used as a negative control, and HeLa cells stimulated with 20 ng/ml TRAIL were used as a positive control for the TRAIL neutralizing antibody. Error bars indicate standard deviations calculated by three independent experiments and the double asterisks (**) represent p<0.01.

To investigate whether induction of TRAIL after stimulation with AP-PG can activate these caspases, the activities of caspases were monitored using Caspase-Glo Assay (Promega). In this experiment, the supernatant of Mono Mac 6 cells was used. The fold induction of TRAIL mRNA in Mono Mac 6 cells was weaker than RAW264.7 cells and macrophage-differentiated THP-1 cells (Figs 1 and 2). However, in Mono Mac 6 cells, when TRAIL mRNA expression was peaked, expression of TNF-α mRNA was decreased to the basal level (Fig 2A). Stimulation with TNF-α is known to be able to activate caspase-8 and -9 as similar with TRAIL stimulation. In addition to TNF-α, we previously reported that other cytokines including IL-1β mRNA is induced after stimulation with AP-PG. IL-1β is known to be involved in the activation of NF-κB and c-jun N-terminal kinase (JNK) [30], and these signaling molecules are crucial for modulating apoptosis induction. In Mono Mac 6 cells, IL-1β mRNA induction was decreased at 24 hours after stimulation with AP-PG as similar as that of TNF-α (data not shown), whereas in other macrophage cell lines, IL-1β mRNA was abundantly expressed when the TRAIL mRNA expression was peaked. Thus, the supernatant of Mono Mac 6 cells was used for the assay. Many cytokines are known to be involved in the induction of other cytokines through the activation of NF-κB signaling pathway. To avoid the influence of other inflammatory cytokines induced after stimulation with AP-PG, we used a protein synthesis inhibitor, cycloheximide. Here HeLa cells were treated with the supernatant of the growth medium from AP-PG-stimulated Mono Mac 6 cells and the activities of the caspases were monitored. The results show that the activity of capsase-8 was not significantly changed in the HeLa cells directly stimulated with the β-glucan (Fig 3A, compare the first and second bars from the left), whereas the supernatant of growth medium from AP-PG-stimulated Mono Mac 6 cells significantly activated caspase-8 (Fig 3A, compare third and fourth bars from the left). Furthermore, this activation of caspase-8 was significantly inhibited by the pre-treatment of the supernatant with anti-TRAIL neutralizing antibody (Fig 3A, compare the fourth and fifth bars from the left). Although caspase-8 activation by the pretreatment with anti-TRAIL neutralizing antibody was not completely inhibited like it was with that of the inhibition against recombinant TRAIL (Fig 3A, compare the fourth to seventh bars from the left), the results show that induction of TRAIL in Mono Mac 6 cells after the stimulation with AP-PG is involved in the activation of caspase-8 to the other cells.

Similar results were obtained in the caspase 9 activation. As shown in Fig 3B, caspase 9 in HeLa cells was activated after the treatment with the supernatant of the cultured medium obtained from the β-glucan stimulated Mono Mac 6 cells (Fig 3B, compare the third and fourth bars from the left). In addition, the activation of caspase 9 is significantly inhibited by the treatment with the TRAIL neutralizing antibody (Fig 3B, compare the fourth and fifth bars from the left). These results demonstrate that TRAIL produced from the Mono Mac 6 cells after the stimulation with AP-PG can induce apoptosis in the HeLa cells, and this may suggest that stimulation of macrophages with AP-PG is able to induce TRAIL dependent apoptosis in tumor cells.

### Difference of TRAIL induction activity between curdlan and AP-PG

**Fig 4:**
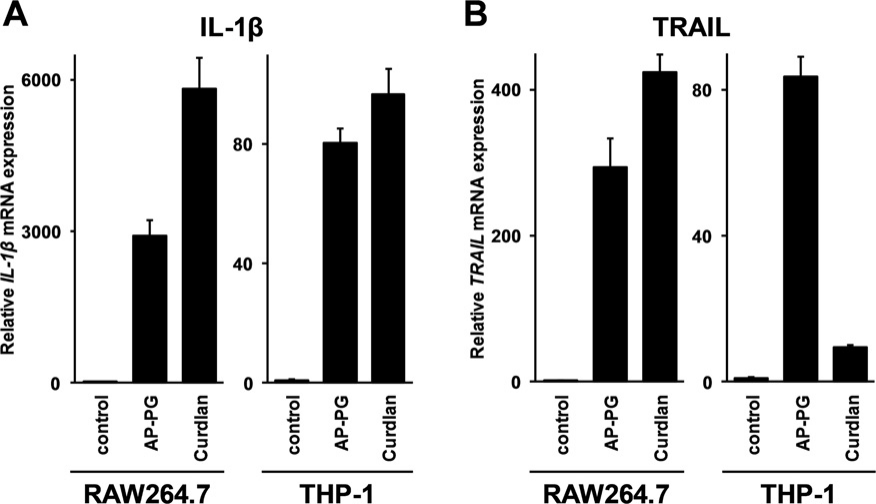
Difference of TRAIL induction activity between curdlan and AP-PG. (A, B) RAW264.7 cells or macrophage-differentiated THP-1 cells were stimulated with curdlan or AP-PG at a concentration of 100 μg/ml. After a 6 hour post-stimulation incubation period, the cells were harvested, and the expression of IL-1β (A) or TRAIL (B) mRNA was monitored by real-time RT-PCR. The data represent relative expression values compared with the mRNA expression in the control cells after normalization with the GAPDH mRNA expressions. The error bars indicate standard deviations calculated from three independent experiments.

To compare TRAIL induction activity of AP-PG with a structurally distinct β-glucan consisting of β-(1,3)-linked glucose as the main chains, curdlan which is known to be a bacteria-derived β-glucan was used. Curdlan is a linear polysaccharide consisting of β-(1,3)-linked glucose, and has almost no branches. As shown in Fig 4A, the results show that stimulation with both curdlan and AP-PG significantly induced IL-1β mRNA in both RAW264.7 and THP-1 cells differentiated into macrophages. Induction of TRAIL mRNA in the macrophage-differentiated THP-1 cells after stimulation with curdlan was significantly weaker than that of AP-PG (Fig 4B). These results would suggest that the efficiency of TRAIL induction is different among β-(1,3)-linked glucans derived from different organisms in a cell type specific manner.

## Discussion

In this report, we demonstrate that stimulation with AP-PG increases TRAIL expression in mouse and human macrophage-like cell lines, RAW264.7 cells, Mono-Mac-6 cells, and macrophage differentiated THP-1 cells. It is also demonstrated that treatment with the supernatant of the growth medium from Mono Mac 6 cells stimulated with AP-PG activates caspase-8 and -9 in HeLa cells; and at least partially, TRAIL secreted into the supernatant is involved in the activation of the caspases.

It is not established whether the TRAIL induction is a primary response to AP-PG stimulation. The results of the time course analysis of the TRAIL mRNA expression demonstrate that the expression of TRAIL mRNA is significantly increased as early as 3 hours and peaked at 6 hours after stimulation with AP-PG in RAW264.7 cells (Fig 1A). On the other hand, TNF-α mRNA expression level is peaked at 3 hours after stimulation with AP-PG (Fig 1B). Although there is remaining a possibility that TRAIL is also induced by primary response to stimulation with AP-PG, these observations suggest that induction of TRAIL is mainly depending on secondary response, such as paracrine and autocrine effects of cytokines produced after stimulation with AP-PG. The time course expression pattern of TRAIL was slower in the Mono Mac 6 cells than in the RAW264.7 cells (Fig 2A). Although the molecular mechanism for the differences in the TRAIL expression pattern is not established, it appears possible that unknown factors are constitutively expressed or activated in RAW264.7 cells, but not in Mono Mac 6 cells, and that these are involved in AP-PG-induced TRAIL expression. In addition, the differences in the time course of the TRAIL expression suggest that the response to AP-PG stimulation is different among macrophage lineages. Therefore, to analyze the differences in TRAIL induction in response to stimulation of AP-PG in various macrophage lineages could be important for a better understanding of the effects of AP-PG on the health of organisms.

The TRAIL mRNA was significantly induced after stimulation with AP-PG in various cell lines, and in all experiments shown in this study. However, the induction levels of TRAIL mRNA were variable in experiments, and also in the same cell line. The reason for these variable induction activities of TRAIL mRNA after stimulation with AP-PG is unknown. The significance of phagocytosis-mediated cell internalization on the recognition of β-glucans followed by activation of immune response was reported in other organism-derived β-glucans [31]. As similar with other organism-derived β-glucans, internalization of AP-PG into the cell is thought to be important for activation of signaling pathway required for TRAIL induction. In macrophages, phagocytosis is known to be altered by various stimuli and cell conditions, and this might be related in the variability of TRAIL induction after stimulation with AP-PG.

As shown in Fig 4, TRAIL induction activity is different between curdlan and AP-PG in the cell-type dependent manner. Two major receptors for β-glucans consisting of β-(1,3)-linked main chains have been identified. One is CR3 (complement receptor 3; also called Mac-1, integrin αM β2) consisting of CD11b and CD18 [32, 33], and the other is dectin-1 (dendritic cell-associated C-type lectin-1) [34]. Previous report has demonstrated that both soluble and particulate β-glucans are able to bind to dectin-1, but dectin-1 mediated signaling pathway is not activated by soluble β-glucans [34, 35]. Curdlan is a particulate β-glucan [35, 36], whereas AP-PG is water soluble [8, 9]. Futher, it has been reported that AP-PG does not exhibit binding activity to dectin-1 [37], while curdlan is known to be an agonist for dectin-1 and is recognized by dectin-1 [38, 39]. The results of our preliminary experiments using antagonistic antibodies against CR3 and dectin-1 showed that the TRAIL induction after stimulation with AP-PG is mainly dependent on CR3. However, the results of gene knockdown experiments using siRNAs specifically silencing CR3 and dectin-1 indicated that not only CR3 but also dectin-1 knockdown strongly inhibited TRAIL induction after stimulation with AP-PG (data not shown). These observations may suggest that difference in dectin-1 binding affinities between curdlan and AP-PG is related in the differences in the induction efficiency of TRAIL, and the crosstalk of CR3 and dectin-1 signaling pathway would be important for the induction of TRAIL.

It is not clarified what factor(s) is/are involved in TRAIL induction after stimulation with AP-PG. A previous report demonstrated that CD11b which is known to be a subunit of CR3 responsible for the recognition of β-glucans [33] is related in TRAIL induction through activation of NF-κB [40]. This may suggest that activation of NF-κB is thought to be involved in TRAIL induction after stimulation with AP-PG. However, activation of NF-κB is also related in induction of TNF-α [41]. Stimulation with LPS is known to activate NF-κB through activation of TLR4-mediated signaling pathway [42]. As shown in Fig 1E and 1F, both AP-PG and LPS stimulations significantly induce TRAIL in RAW264.7 cells, on the other hand, induction of TNF-α after stimulation with AP-PG is apparently weaker than that of LPS. Molecular mechanism for these different induction patterns of death ligands between AP-PG and LPS still remains unclear. Selectivity of NF-κB subunits activated after stimulation with AP-PG might be involved in this effective induction of TRAIL [43].

Thus far, we demonstrated that stimulation with AP-PG effectively induces TRAIL in macrophages. Here, we propose a possibility that TRAIL induced in macrophages after the stimulation with AP-PG is involved in the anti-tumor activity of AP-PG. Originally, TRAIL was discovered as a cytokine which induces apoptosis in transformed cells, but not in most types of untransformed cells [17]. However, TRAIL functions are not restricted to tumor killing in mammals. Previously, the importance of TRAIL in the suppression of autoimmune lymphocyte activity has been demonstrated [44, 45]. In addition, a previous study using recombinant TRAIL demonstrated that activation of the TRAIL-mediated signaling pathway is effective in the treatment of autoimmune encephalomyelitis [46]. Efficacy to experimental autoimmune encephalomyelitis was also reported for zymosan, a β-glucan derived from the yeast cell wall [47]. Therefore, induction of TRAIL after stimulation with β-glucans may be involved in the molecular mechanisms that exhibit efficacy to experimental autoimmune encephalomyelitis. The *A*. *pullulans*-cultured fluid containing AP-PG as a main component is consumed as a food supplement. It is not clarified whether orally administered AP-PG exhibits TRAIL induction activity for macrophages localized in small intestine and other organs. A　previous report demonstrated that orally administered β-glucan is able to activate lymphocytes localized in Peyer's patch [48]. In addition, effects of orally administered β-glucan for activation of alveolar macrophages have been reported [49]. Therefore, there is a possibility that orally administered AP-PG exhibits activation of immune system including NK cell activity through TRAIL induction. Further investigations are required to understand the significance of the TRAIL induction after stimulation with β-glucans including AP-PG

## Materials and Methods

### Cell culture and preparation of the purified β-glucan produced by *A*. *pullulans*

The RAW264.7 (ATCC TIB-71) [18] and THP-1 cells (ATCC TIB-202) [20] were grown and maintained in RPMI 1640 medium supplemented with 10% fetal bovine serum, 100 U/ml penicillin, 100 mg/ml streptomycin; and Mono Mac 6 cells (DSMZ ACC 124) [19] were cultured in RPMI1640 medium supplemented with 10% fetal bovine serum, 100 U/ml penicillin, 100 mg/ml streptomycin, 1% non-essential amino acids (Life Technologies), and 1% OPI media supplement (Sigma-Aldrich). These cells were grown at 37°C in 5% CO2 in a humidified incubator. To differentiate THP-1 cells into macrophages, THP-1 cells were treated with phorbol-12-myristate-13-acetate (PMA) at the concentration of 100 nM for 72 hours. The cells were incubated in growth medium without PMA for an additional 24 hours, and used in the study. Normal human monocytes were purchased from commercially available products (Promocell, Heidelberg, Germany). The cells were differentiated into macrophages using a commercially available medium (M1-Macrophage Generation Medium DXF; Promocell) in accordance with the manufacturer's protocol, and used in the study. The procedure for preparation of the purified AP-PG from *A*. *pullulans* cultured fluid was described elsewhere [15]. Curdlan derived from *Alcaligenes faecalis* was obtained from commercially available products (Sigma-Aldrich).

### Real-Time RT-PCR

Total RNA from the cells was isolated using Trizol reagent (Invitrogen, Carlsbad, CA). The isolated total RNAs were treated with DNaseI (Takara, Otsu, Japan) and then subjected to oligo-dT- and random-primed reverse transcription using ReverTra Ace (Toyobo, Osaka, Japan). Real-time RT-PCR was performed using SYBR Premix Ex Taq (Takara). The PCR reactions and analysis of mRNA expressions were performed using the CFX96 Real-Time PCR Detection System (Bio-Rad, Hercules, CA). Each procedure was performed according to the manufacturer's protocol. The sequence of specific primer set for amplification of mouse TNF-α is described elsewhere [50], and the following specific primer sets were designed and used in this study: Mouse FasL (sense primer: 5’- CCACCTGCAGAAGGAACTGGCA -3’; anti-sense primer: 5’- ATGGGCCACACTCCTCGGCT -3’); mouse TRAIL (sense primer: 5’- CCAACGAGATGAAGCAGCTGCAGG -3’; anti-sense primer: 5’- CCTGCAAGCAGGGTCTGTTCAAGA -3’); mouse IL-1β (sense primer: 5’- GCCTCGTGCTGTCGGACC -3’; anti-sense primer: 5’- TGTCGTTGCTTGGTTCTCCTTG -3’); human TNF-α (sense primer: 5’- CCCAGGGACCTCTCTCTAATC -3’; anti-sense primer: 5’- ATGGGCTACAGGCTTGTCACT -3’); human FasL (sense primer: 5’- CTTGGTAGGATTGGGCCTGG -3’; anti-sense primer: 5’- TGTGTGCATCTGGCTGGTAG -3’); human TRAIL (sense primer: 5’- CCGGCTGCCTGGCTGACTTAC -3’; anti-sense primer: 5’- TCAGCACGCAGGTCTGTCCC -3’); human IL-1β (sense primer: 5’- GCAGCCATGGCAGAAGTACCTGA -3’; anti-sense primer: 5’- CCAGAGGGCAGAGGTCCAGGTC -3’).

### Western blotting analysis

RAW264.7 or Mono Mac 6 cells were stimulated with AP-PG at a concentration of 100 μg/ml for 24 hours. For inhibition of TRAIL secretion, Mono Mac 6 cells were treated with 10 μg/ml of Brefeldin A (Sigma-Aldrich) for 3 hours before the end of stimulation. After the stimulation, the cells were collected, washed once with phosphate-buffered saline, and then lysed with RIPA buffer (radioimmune precipitation buffer; 25 mM Tris-HCl [pH 7.5], 150 mM NaCl, 1% Nonidet P-40, 1% sodium deoxycholate, 0.1% sodium dodecyl sulfate [SDS]) supplemented with protease inhibitor (Complete Mini; Roche). After the cell debris was removed by centrifugation, 20 μg protein of each cell extract was subjected to SDS polyacrylamide gel electrophoresis, and blotted onto a polyvinyl difluoride (PVDF) transfer membrane (Immobilon-P; Millipore, Bedford, MA). Soluble and membrane bound forms of TRAIL proteins were detected using an anti-TRAIL specific antibody (Oriental Yeast, Tokyo, Japan), and an anti-β-actin antibody (Chemicon, Temecula, CA) was used as a loading control.

### Monitoring activation of caspases

Activation of caspases was monitored using a Caspase Glo Assay kit (Promega, Madison, WI) with a luminometer (Mithras LB940; Berthold, Bad Wildbad, Germany) in accordance with the manufacturer's instructions. A protein synthesis inhibitor, cycloheximide (Sigma-Aldrich) was used to suppress the activation of NF-κB followed by induction of cytokines. Anti-TRAIL neutralizing antibody (Oriental Yeast, Tokyo, Japan) was used for inhibition of TRAIL activity, and recombinant TRAIL (Super Killer TRAIL; Enzo Life Sciences, Farmingdale, NY) was used as a positive control for monitoring activation of caspases and activity of neutralizing antibody.

### Statistical analysis

To check for significant differences between the indicated pairs of data, a two-tailed unpaired Student's t-test was performed in this study.
